# White Matter Changes in HIV+ Women with a History of Cocaine Dependence

**DOI:** 10.3389/fneur.2017.00562

**Published:** 2017-10-31

**Authors:** Kathryn-Mary Wakim, Ciara J. Molloy, Ryan P. Bell, Lars A. Ross, John J. Foxe

**Affiliations:** ^1^The Cognitive Neurophysiology Laboratory, The Del Monte Institute for Neuroscience, Department of Neuroscience, University of Rochester, Rochester, NY, United States; ^2^Department of Psychiatry and Behavioral Sciences, Duke University School of Medicine, Durham, NC, United States; ^3^The Sheryl and Daniel R. Tishman Cognitive Neurophysiology Laboratory, Department of Pediatrics, Albert Einstein College of Medicine and Montefiore Medical Center, Bronx, NY, United States; ^4^The Sheryl and Daniel R. Tishman Cognitive Neurophysiology Laboratory, Department of Neuroscience, Albert Einstein College of Medicine and Montefiore Medical Center, Bronx, NY, United States

**Keywords:** diffusion tensor imaging, white matter, human immunodeficiency infection, cocaine, abstinence, addiction

## Abstract

Cocaine use is associated with the transmission of human immunodeficiency (HIV) virus through risky sexual behavior. In HIV+ individuals, cocaine use is linked with poor health outcomes, including HIV-medication non-adherence and faster disease progression. Both HIV and cocaine dependence are associated with reduced integrity of cerebral white matter (WM), but the effects of HIV during cocaine abstinence have not yet been explored. We used diffusion tensor imaging (DTI) to understand the effect of combined HIV+ serostatus and former cocaine dependence on cerebral WM integrity. DTI data obtained from 15 HIV+ women with a history of cocaine dependence (COC+/HIV+) and 21 healthy females were included in the analysis. Diffusion-based measures [fractional anisotropy (FA), radial diffusivity (RD), mean diffusivity, and axial diffusivity] were examined using tract-based spatial statistics and region-of-interest analyses. In a whole-brain analysis, COC+/HIV+ women showed significantly reduced FA and increased RD in all major WM tracts, except the left corticospinal tract for RD. The tract with greatest percentage of voxels showing significant between-group differences was the forceps minor (FA: 75.6%, RD: 59.7%). These widespread changes in diffusion measures indicate an extensive neuropathological effect of HIV and former cocaine dependence on WM.

## Introduction

Simultaneous human immunodeficiency (HIV) infection and cocaine use poses a personal and public health crisis. A recent multicenter study demonstrated that HIV+ females who are persistent users of crack cocaine are 3.6 times more likely to die from AIDS-related complications than their HIV+ non-using counterparts (1). Although more men use drugs than women (2), women demonstrate more severe drug use (3) and progress from initiation to dependence faster than men (4). Women also show the fastest growing rates of new HIV infections, from 8% in 1985 to 25% in 2011 (5). In both men and women, cocaine use is associated with a faster transition from HIV to AIDS (6), increased rates of neurocognitive impairment (7), and contributes to continued propagation of HIV through risky sexual behavior (8, 9). Although the introduction of antiretroviral therapy has greatly reduced the impact of HIV on the central nervous system, neurocognitive impairment continues to present a widespread problem (10).

Recent evidence suggests that the co-occurrence of both conditions can result in neuropathological synergy, with cocaine-abusing HIV+ individuals demonstrating more severe neural and behavioral deficits than individuals with either condition alone. For example, current cocaine abuse in HIV is associated with significantly faster disease progression, even after controlling for HIV-medication non-compliance (6). While the molecular mechanisms involved in the interaction of cocaine and HIV are not well understood, synergistic effects have been demonstrated. Cocaine has been found to act in concert with HIV proteins to promote disruption of the blood–brain barrier (11). Increased extracellular dopamine, a consequence of acute cocaine exposure, has also been shown to promote viral replication in infected macrophages (12).

Substantial white matter (WM) changes have been demonstrated in individuals with HIV+ serostatus alone (13, 14) and current cocaine dependence alone (15–17) using diffusion tensor imaging (DTI). DTI is a non-invasive neuroimaging technique used to identify WM microstructural changes *in vivo* and provides an indirect measure of WM integrity *via* quantification of water diffusion parameters within neural tracts (18). Neuropathological changes in DTI metrics are associated with reduced cognitive function in HIV+ individuals (14), as well as poorer treatment outcome in cocaine dependence (17). In one study, HIV-associated WM impairments in the DTI measure of mean diffusivity (MD) were found to be more severe in individuals with a history of substance abuse (19), providing preliminary evidence for a synergistic interaction of former substance abuse and HIV on WM pathology. However, another recent DTI study investigating the combined effect of current cocaine abuse and HIV on WM did not show a synergistic effect (20). In that study, Cordero and colleagues found no effect of current cocaine dependence on WM in both HIV+ and HIV− subjects (20), in contrast to a growing body of literature demonstrating WM impairment in current cocaine dependence (15–17).

Given that WM mediates communication between brain regions, integrity of these tracts is essential for optimal brain functioning. The goal of the current research was to investigate the combined effect of HIV+ serostatus and former cocaine dependence on WM using DTI in a cohort of HIV+ women with a history of cocaine dependence (COC+/HIV+). As an exploratory aim, we also investigated the relationship between DTI metrics, disease measures (CD4 count, duration of abstinence), and self-reported impulsivity. We hypothesized that HIV+ serostatus in women with a history of cocaine dependence would be associated with global neuropathological reductions in measures of WM integrity. Although our study could not specifically address the possibility that WM changes may be more severe in COC+/HIV+ women compared with women with HIV+ serostatus alone or former cocaine dependence alone, our results comparing COC+/HIV+ to healthy controls may still yield novel insights into the neuropathogensis of HIV in this vulnerable population.

## Materials and Methods

### Subjects

16 HIV+ women with a history of cocaine dependence (COC+/HIV+) and 21 healthy females participated in the study. Abstinence from illegal drugs was determined by self-report corroborated by a urine test on the scan date. Patients were recruited from clinics associated with the Einstein-Montefiore Center for AIDS research, who verified past cocaine dependence and HIV+ status. Healthy controls were recruited from a university-wide database for research study participation. Potential participants completed an in-person screening and returned on a different day for scanning. Groups differed in age [*t*_(34)_ = 3.91, *p* < 0.001], years of education [*t*_(34)_ = 4.76, *p* < 0.001], and ethnicity (Fisher’s exact count data, *p* < 0.001) (Table 1). As age (21) and years of education (22), key components of socioeconomic status (23), are known to impact brain structure, both variables were included as covariates in all subsequent analyses. Since no research has demonstrated ethnicity-related WM differences between African-American, Caucasian, and/or Hispanic individuals, ethnicity was not included as a covariate. Exclusion criteria for the study were as follows: (1) head trauma resulting in loss of consciousness for longer than 30 min, (2) the presence of any past or current brain pathology, (3) a positive urine screening for any illegal drugs, and (4) the presence of any contraindication to MRI scanning. Healthy controls were excluded for any self-reported history of psychiatric illness or a history of drug or alcohol dependence. Due to high rates of psychiatric comorbidity in HIV+ individuals, COC+/HIV+ were excluded for the presence of an untreated psychiatric condition. 3 COC+/HIV+ participants had a diagnosis of major depressive disorder (one with comorbid generalized anxiety disorder), and one had a diagnosis of bipolar disorder.

**Table 1:** Demographics and subject characteristics.

	Group		*t*-Value	*p*-Value
	COC+/HIV+ (*n* = 15)	Healthy (*n* = 21)		
Age	50.3 ± 5.42	36.3 ± 13.0	3.91	<0.001
Years of education	12.1 ± 2.37	16.8 ± 3.24	4.76	<0.001
Ethnicity (Black:White:Hispanic)	12:1:2	2:10:8^a^	–	<0.001^b^
Duration abstinence (weeks)	460 ± 381	–	–	–
Years of cocaine use	11.94 ± 6.93	–	–	–
CD4 (cells/μL)	609 ± 265	–	–	–
Viral load				
% of HIV+ subjects with <75 copies/mL	66%	–	–	–
BIS-11 Total Score	64.5 ± 8.74	51 ± 8.25	3.86	<0.001

*Values are presented as mean ± SD*.

*Two-tailed independent samples t-tests were performed to determine the significance of between-group differences*.

*^a^Ethnicity of n = 1 subject is not known*.

*^b^Fisher’s exact test*.

Participants with a history of cocaine dependence completed the cocaine subsection of the Kreek–McHugh–Schluger–Kellogg (KMSK) Scale to further characterize cocaine dependence during the most severe period of use (24). Because of the high rates of alcohol and other drug comorbidity among COC+/HIV+ populations, patients were not excluded if they had abused other drugs or alcohol prior to the onset of their cocaine abstinence as long as cocaine was identified as their primary drug of choice based on referral from addiction treatment centers, the KMSK, and verbal agreement. 2 COC+/HIV+ individuals had past comorbid alcohol abuse, and 1 COC+/HIV+ individual had comorbid past heroin dependence.

COC+/HIV+ participants were abstinent from cocaine for an average of 460 ± 381 weeks (range: 8.7–1,043 weeks). Years of self-reported cocaine use was collected from 11 of the 15 participants included in the final analysis; patients used cocaine for an average of 11.94 ± 6.53 years (range: 3–22). All COC+/HIV+ participants were receiving highly active antiretroviral therapy. 1 COC+/HIV+ patient had an additional diagnosis of hepatitis C. Current CD4 count was available for 13 out of the 15 women included in the final sample. These patients had an average current CD4 count of 609 ± 265 cells/μL (range: 197–1,097). Viral load data were available for 12 out of the 15 HIV+ participants included in the final sample, and 66% of these patients had a current viral load below 75 copies/mL (range: undetected—25,027). No COC+/HIV+ patients had any AIDS-defining clinical conditions. This study was approved by the Albert Einstein College of Medicine institutional review board. Written, informed consent was obtained from all participants in accordance with the tenants of the Declaration of Helsinki.

### Behavioral Measures

Impulsive was assessed through self-administration of the Barratt Impulsiveness Scale (BIS-11). BIS-11 scores were available for 14 COC+/HIV+ participants and 10 healthy controls (25). This measure contains 30 questions aimed to assess personality traits associated with risk taking and impulsivity. Subsections include questions pertaining to attention impulsiveness, motor impulsiveness, and non-planning impulsiveness.

### Scanning Procedure

MRI was performed at the Magnetic Resonance Research Center at the Albert Einstein College of Medicine using a 3.0 T Freewave Achieva MRI Scanner (Philips Medical Systems, Best, The Netherlands) with a 32-Channel SENSE RF head coil (3 MHz per channel bandwidth; 80 MHz per channel all digital sampling rate). A T1-weighted, sagittal 3D MPRAGE acquisition over a 240 mm field of view with 240 × 240 in-plane matrix and 1 mm isotropic resolution, TR = 8.2 s/TE = 3.7 ms, SENSE factor = 2 (3 min) was acquired. Diffusion-weighed images were obtained using single shot echo-planar imaging (EPI) along 32 independent, non-collinear diffusion sensitizing directions at *b* = 800 s/mm^2^ (TR = 7,600 ms, TE = 56 ms, voxel size: 2 mm × 2 mm × 2 mm, FOV = 256 mm^2^, in-plane imaging matrix = 128 mm × 127 mm, number of slices = 70, and SENSE factor = 2.5). Two additional images with no diffusion weighting (*b* = 0) were also collected. Total DTI scan time was 4 min·26 s.

### DTI Processing

Data were preprocessed using the ExploreDTI Software (v 4.8.6)^1^ (26). In one step, the data were corrected for eddy current-induced geometric distortions, subject motion, and EPI by coregistration and resampling to each subjects T1-weigthed anatomical image (27). The B-matrix rotation was also performed in this step to reorient the data appropriately (28). After visual inspection of the data, one COC+/HIV+ participant was removed due to excessive motion artifacts in the B0 image which persisted after motion correction. Therefore, 15 COC+/HIV+ and 21 healthy control subjects were included in the analysis. Diffusion-based measures of fractional anisotropy (FA), radial diffusivity (RD), axial diffusivity (AD), and MD were extracted for each participant.

### Data Analysis

Voxelwise statistical analyses were carried out using tract-based spatial statistics from FMRIB’s Software Library^2^ (29, 30). Following preprocessing, FA maps from ExploreDTI were aligned to a 1 mm × 1 mm × 1 mm standard space target image (FMRIB58_FA) using FMRIB’s non-linear image registration tool. The target image was then aligned to Montreal Neurological Institute (MNI) template space using an affine transformation. Each subjects’ FA images were transformed to MNI space by combining the non-linear transformation to the target FA image with the affine transformation to the MNI template in one step to avoid resampling the image twice. A mean FA map was created by averaging all subjects’ FA images; this map was then thinned (threshold of 0.2) to generate a mean FA skeleton representing the center of the tracts common to all groups. Each subject’s aligned FA images were then projected onto this FA skeleton for voxelwise analyses. The same non-linear transformation and FA skeleton projection procedure used for FA were applied to the other diffusion measures (RD, AD, and MD).

Group comparisons for FA, MD, AD, and RD diffusion measures were conducted using the FMRIB randomize toolbox. Ten thousand permutations were used to estimate a null distribution for each contrast (control > COC+/HIV+ and COC+/HIV+ > control) with age and years of education included as covariates. The results were thresholded at *p* ≤ 0.05 and corrected for multiple comparisons across voxels using the threshold-free cluster enhancement option in randomize (31). To further quantify these results, the percentage of “affected” voxels (those showing significant between-group differences in diffusion metrics) was calculated for each major WM tract. WM tracts were identified using John’s Hopkins University (JHU) DTI-based WM atlas. The mean FA skeleton was masked with JHU atlas-derived regions of interests (ROI). The percentage of affected voxels in each ROI was then computed by dividing the number of WM voxels showing significant between-group differences by the total number of WM voxels in each ROI.

The average FA and RD value from WM voxels were also extracted from cognitive control-related WM tracts for correlations with BIS-11 score. Subjects’ FA skeletons were masked with the JHU atlas-derived ROI for the forceps minor and bilateral uncinate fasciculus. For each subject, the average FA or RD value of voxels within these masks were extracted.

Between-group differences in demographics were examined using two-tailed independent samples *t*-tests conducted in R statistical computing software (32). Pearson’s correlations between diffusion measures, CD4 count and duration of abstinence were performed. Partial correlations with mean FA and RD values from the forceps minor and bilateral uncinate fasciculus were performed using the R package “ppcor” with age and years of education included as covariates (33).

To measure possible between-group differences in brain volume, total estimated intracranial volume was calculated based on T1-weighted images using FreeSurfer^3^ (34). A two-tailed independent samples *t*-test was performed.

## Results

### Behavioral Results

COC+/HIV+ subjects scored significantly higher on the BIS-11 measure of self-reported impulsivity compared with healthy controls [*t*_(22)_ = 3.86, *p* < 0.001].

### Brain Measures

**Figure 1:**
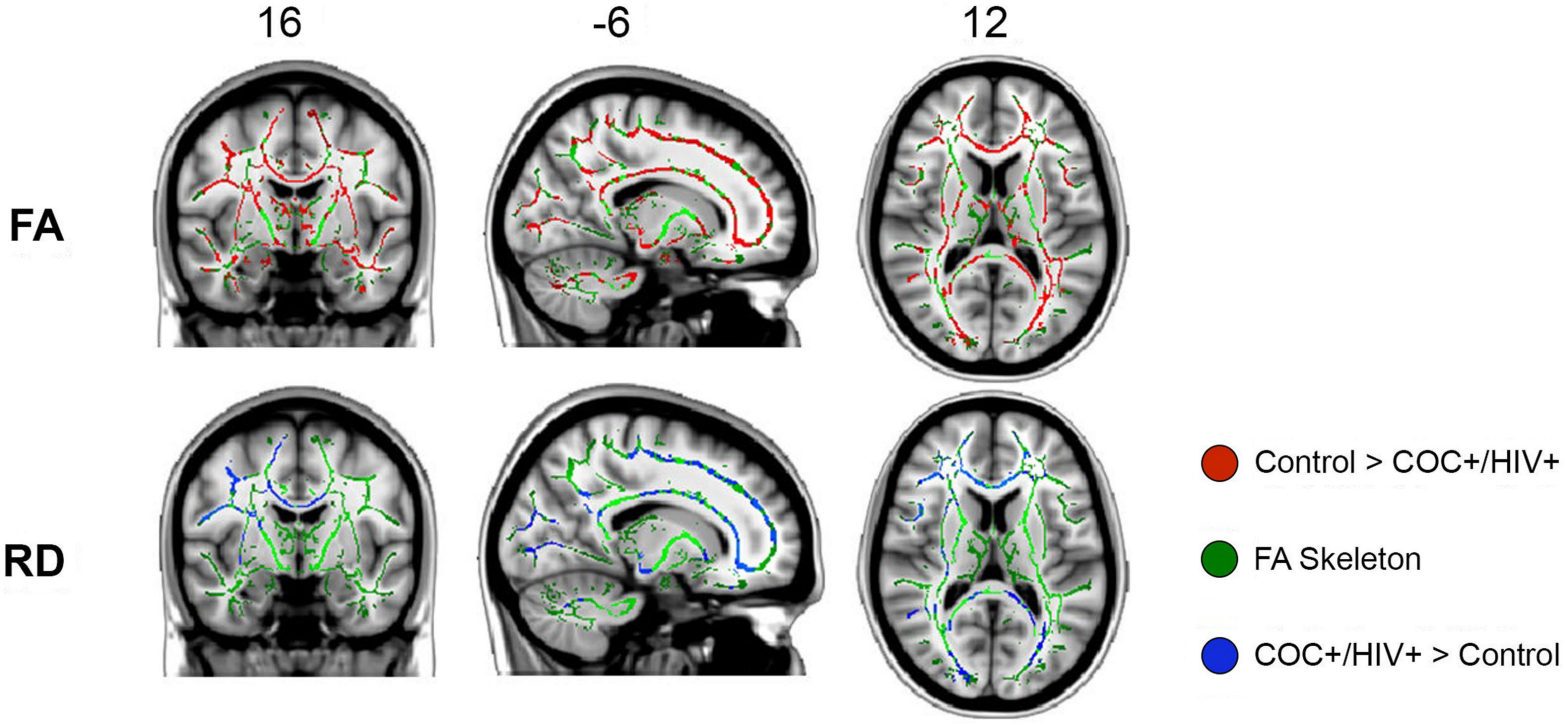
Tract-based spatial statistics results: fractional anisotropy (FA) and radial diffusivity (RD) white matter regions showing statistically significant decreases in FA (red) and increases in RD (blue) in COC+/HIV+ subjects compared with controls. All images are presented in radiological convention.

Statistically significant between-group differences were seen diffusely in WM tracts for FA and RD diffusion measures such that decreased FA and increased RD were found in COC+/HIV+ patients compared with controls (Figure 1; Table 2). No WM regions showed decreased FA or increased RD in controls compared with COC+/HIV+. There were no statistically significant voxelwise differences between groups for diffusion measures of MD and AD.

**Table 2:** Peak voxels.

Region	Measure	Contrast	Peak *t*-value	*X*	*Y*	*Z*
Body of corpus callosum	FA	Control > COC+/HIV+	5.64	8	−9	27
	RD	COC+/HIV+ > Control	5.33	8	−9	27
	RD	COC+/HIV+ > Control	5.32	3	−10	26
Genu of corpus callosum	FA	Control > COC+/HIV+	4.90	−3	24	14
	FA	Control > COC+/HIV+	5.0	11	31	10
	RD	COC+/HIV+ > Control	4.28	12	33	1
R. postcentral gyrus white matter (WM)	FA	Control > COC+/HIV+	5.5	34	−28	45
	RD	COC+/HIV+ > Control	5.21	33	−28	46
R. interior fronto-occipital fasciculus	RD	COC+/HIV+ > Control	4.66	38	34	9
R. superior longitudinal fasciculus	RD	COC+/HIV+ > Control	4.57	27	−52	38
R. superior temporal WM	FA	Control > COC+/HIV+	7.7	54	−25	7

*Voxels showing statistically significant between-group differences in FA and RD. X, Y, and Z values are presented as Montreal Neurological Institute coordinates*.

*FA, fractional anisotropy; RD, radial diffusivity*.

The percentage of affected voxels calculation showed that the between-group differences spanned all major WM tracts for FA and most WM tracts for RD (Table 3). The tract with the greatest percentage of voxels showing significant between-group differences was the forceps minor (FA: 75.6%, RD: 59.7%). For FA, the region with the lowest percentage of voxels showing significant differences was the right cingulum (31.6%). For RD, there were no significant between-group differences on the left corticospinal tract.

**Table 3:** Percentage of voxels in major WM tracts showing significant between-group differences.

Tract name	L/R	Affected voxels (FA)	Affected voxels (RD)	Total voxels	Percent affected (FA)	Percent affected (RD)
Anterior thalamic radiation	Left	2,343	398	4,662	**50.3**	**8.54**
	Right	2,112	1,260	3,986	**53.0**	**31.6**
Cingulum	Left	605	224	1,024	**59.1**	**21.9**
	Right	162	10	513	**31.6**	**1.95**
Corticospinal	Left	1,072	0	2,861	**37.5**	**0**
	Right	1,338	689	2,800	**47.8**	**24.6**
Forceps major	Bilateral	2,482	1,632	4,119	**60.3**	**39.6**
Forceps minor	Bilateral	4,145	3,277	5,485	**75.6**	**59.7**
Inferior fronto-occipital fasciculus	Left	3,339	875	5,361	**62.3**	**16.3**
	Right	3,130	2,762	5,653	**55.4**	**48.9**
Inferior longitudinal fasciculus	Left	2,458	325	4,319	**56.9**	**7.52**
	Right	1,825	1,092	3,422	**53.3**	**31.9**
Uncinate fasciculus	Left	1,211	405	2,072	**58.4**	**19.5**
	Right	604	423	968	**62.4**	**43.7**

*Affected voxels represent those showing significant decreases in FA or increases in RD in COC+/HIV+ compared with controls*.

*FA, fractional anisotropy; RD, radial diffusivity; WM, white matter*.

There was no significant difference in estimated total intracranial volume between groups [*t*_(34)_ = 1.055, *p* = 0.30], therefore it was not included as a covariate in any analyses.

### Correlations

To investigate the relationship between diffusion metrics and disease measures, the average FA and RD value across all WM voxels was extracted. The average FA and RD values were correlated with duration of abstinence (FA: *r* = 0.18, *p* = 0.51; RD: *r* = −0.19, *p* = 0.47) and CD4 count (FA: *r* = −0.06, *p* = 0.84; RD: *r* = 0.11, *p* = 0.72). No significant correlations were found.

Further analyses examined the association between impulsivity and DTI metrics specifically within cognitive control-related brain circuitry. Partial correlations between BIS-11 impulsivity score and the mean FA and RD in the forceps minor (FA: *r* = −0.39, *p* = 0.074; RD: *r* = 0.34, *p* = 0.12), left uncinate fasciculus (FA: *r* = −0.31, *p* = 0.16; RD: *r* = 0.27, *p* = 0.22), and right uncinate fasciculus (FA: *r* = −0.36, *p* = 0.10; RD: *r* = 0.37, *p* = 0.09) showed no statistically significant correlations.

## Discussion

The main finding of this study is that the co-occurrence of HIV+ serostatus and former cocaine dependence is associated with widespread alterations in diffusion measures of WM integrity. Validation of diffusion metrics against animal models implicates decreased FA with non-specific WM impairment, with increased RD (35) and decreased AD (36) indexing demyelination and axonal injury, respectively. Therefore, our results showing reduced FA and increased RD suggest that former cocaine dependence in individuals with HIV may be associated with a pattern of disrupted myelination. Indeed, early autopsy studies show that diffuse myelin pallor, a histopathological feature associated with poorly maintained myelin, is fairly common in individuals with AIDS (37). This feature may also not be restricted to AIDS patients; one autopsy study also demonstrated diffuse myelin pallor in HIV+ non-AIDS patients with former heroin abuse (38).

We observed differences in measures of WM integrity across all major WM tracts. The fact that the greatest WM impairment was seen in the forceps minor, a tract connecting the lateral and inferior portions of the frontal lobe, is consistent with previous DTI findings showing reduced frontal FA in cocaine addiction (16) and HIV (13) separately. Our findings also accord well with previous research demonstrating ACC hypoactivation during verbal learning in COC+/HIV+ women (39) and provide a potential structural explanation for this impairment. Extensive research has also implicated frontal lobe dysfunction with increased behavioral impulsivity and risk of substance dependence (40, 41). Consistent with this idea, we observed significant increases in self-reported impulsivity in COC+/HIV+ compared with controls. That this marker did not correlate with any diffusion metrics may reflect a small sample size.

Literature investigating WM deficits in HIV+ current substance users has also shown correlations between DTI findings and cognitive measures (42). In a recent study, Tang and colleagues demonstrated significant decreases in FA and increases in RD in the genu of the corpus callosum, consistent with the forceps minor, in HIV+ psychostimulant users compared with controls. Across the entire sample, decreased genu FA was related to impaired performance on assessments of sustained attention and attentional set shifting. Although this study did not specifically investigate cognitive function, our findings of global WM impairment in COC+/HIV+ women, with the greatest degree of impairment along frontal tracts, agree with findings from Tang et al., which point to a link between WM impairment and cognitive dysfunction. Those widespread differences in diffusion metrics persist in our cohort despite cocaine abstinence could suggest that HIV is the primary driving factor of the neuropathology. Indeed, this idea is supported by results from a recent study showing no effect of current cocaine use on WM pathology in HIV+ subjects (20). However, it should be noted that Cordero et al.’s recent findings differ from many those of studies demonstrating WM impairment in current cocaine dependence in the absence of HIV (15–17).

Although these results shed light on the neuropathogenesis of HIV in abstinent cocaine-dependent women, this study has a number of limitations. First, as this study does not include patients with former cocaine dependence alone or HIV+ serostatus alone, we are unable to tease apart the individual contributions of either factor to the observed COC+/HIV+ phenotype. In addition, participant groups also differed in age, years of education, ethnicity, and psychiatric comorbidities. Because age and years of education are known to affect WM integrity (21, 22), both variables were included as covariates in all analyses. Ethnicity was not included as a covariate, as no research has demonstrated ethnicity-related WM differences between African-American, Caucasian and/or Hispanic individuals. Four COC+/HIV+ patients had a psychiatric diagnosis; no healthy control subjects had any self-reported history of psychiatric illness. Although all affected patients were undergoing treatment for their condition, psychiatric disorders have been consistently associated with frontal WM impairment (43, 44). In addition, substance abuse has found to be associated with reduced antiretroviral medication non-adherence (7, 45). Although participants in this study were abstinent from all illegal drugs, it is possible that a history of antiretroviral medication non-adherence shaped the course of WM changes in the patient cohort.

The primary strength of this study lies in the uniqueness of its dataset. Women are an understudied and underrepresented population in both the HIV and addiction literature. To our knowledge, our study is the only DTI investigation specifically targeting HIV+ women with a history of drug dependence. Future research should investigate the relationship between impaired diffusion metrics and other clinical outcome variables, including daily functioning, HIV-medication adherence, and measures of cognition to assess whether they may provide a potential mechanistic explanation for HIV-associated cognitive impairments commonly observed in this population.

## Ethics Statement

This study was approved by the Albert Einstein College of Medicine institutional review board. Written, informed consent was obtained from all participants in accordance with the tenants of the Declaration of Helsinki.

## Author Contributions

JF and RB were responsible for initial study concept. RB and LR were responsible for participant recruitment and data collection. K-MW and CM contributed to the data analysis. K-MW, CM, and JF contributed to data interpretation. K-MW wrote the first draft of the manuscript. CM and JF provided extensive editorial input and critical revisions of the manuscript. All the authors reviewed the content of the paper and approved the final version.

## Conflict of Interest Statement

All authors of this paper declare no conflicts of interest, financial or otherwise, that may have biased their contributions to this work.

## Footnotes

^1^www.exploredti.com.

^2^https://fsl.fmrib.ox.ac.uk/fsl/fslwiki.

^3^https://surfer.nmr.mgh.harvard.edu/.
